# Evolution of the Speciation and Mobility of Pb, Zn and Cd in Relation to Transport Processes in a Mining Environment

**DOI:** 10.3390/ijerph17144912

**Published:** 2020-07-08

**Authors:** Intissar Elmayel, José María Esbrí, García-Ordiales Efrén, Eva-María García-Noguero, Zouhair Elouear, Bouzid Jalel, Alessandro Farieri, Nieves Roqueñí, Pablo Cienfuegos, Pablo Higueras

**Affiliations:** 1 Laboratoire Génie Environnement Ecotechnologie, Ecole Nationale d’Ingénieurs de Sfax, Université de Sfax, 3038 Sfax, Tunisia; elouear_zouhair@yahoo.fr (Z.E.); bouzid.jalel@gmail.com (B.J.); 2 Departamento de Ingeniería Geológica y Minera, Instituto de Geología Aplicada, Universidad de Castilla-La Mancha, 13400 Almadén, Spain; JoseMaria.Esbri@uclm.es (J.M.E.); eva.garcia@noguero.es (E.-M.G.-N.); pablo.higueras@uclm.es (P.H.); 3 Departamento de Explotación y Prospección de Minas, Escuela de Ingeniería de Minas, Energía y Materiales de Oviedo, Universidad de Oviedo, 33004 Oviedo, Spain; garciaefren@uniovi.es (G.-O.E.); nievesr@uniovi.es (N.R.); cienfuegospablo@uniovi.es (P.C.); 4 Dipartimento di Scienze della Terra, Università degli studi di Firenze, 50121 Firenze, Italy; alessandro.farieri@stud.unifi.it

**Keywords:** selective extractions, sequential extractions, (bio)availability, potentially toxic elements, Tunisia

## Abstract

Elements in mining extracts can be potentially toxic if they are incorporated into soils, sediments or biota. Numerous approaches have been used to assess this problem, and these include sequential extractions and selective extractions. These two methods have limitations and advantages, and their combined use usually provides a rough estimate of the availability or (bio)availability of potentially toxic elements and, therefore, of their real potential as toxicants in food chains. These indirect speciation data are interesting in absolute terms, but in the work described here, this aspect was developed further by assessing the evolution of availability-related speciation in relation to the transport processes from the emission source, which are mainly fluvial- and wind-driven. This objective was achieved by characterizing tailings samples as the source of elements in soils and sediments at increasing distances to investigate the evolution of certain elements. The standard procedures employed included a sequential five-step extraction and a selective extraction with ammonium acetate. The results show that the highest percentages of Zn and Pb in tailings, soils and sediment samples are associated with oxyhydroxides, along with a significant presence of resistant mineralogical forms. In the case of Cd, its association with organic matter is the second-most important trapping mechanism in the area. The physicochemical mechanisms of transport did not transform the main mineralogical associations (oxyhydroxides and resistant mineralogical forms) along the transects, but they produced a chaotic evolution pattern for the other minor matrix associations for Zn and a decrease in exchangeable and carbonate-bound forms for Pb in soils. Interestingly, in sediments, these mobile forms showed a decrease in Zn and a chaotic evolution for Pb. The most probable reason for these observations is that Zn^2+^ can form smithsonite (ZnCO_3_) or hydrozincite (Zn_5_(CO_3_)_2_(OH)_6_), which explains the retention of a carbonate-bound form for Zn in the soil transect. In contrast, Pb and Cd can appear as different mineral phases. The order of (bio)availability was Pb > Zn > Cd in tailings but Cd > Pb > Zn in soils. The physicochemical processes involved in transport from tailings to soils produce an increase in Cd (bio)availability. The trend is a decrease in bioavailability on moving away from the source (tailings), with maximum values obtained for Cd near to the source area (200–400 m).

## 1. Introduction

Contamination with potentially toxic elements (PTEs) (also described as “heavy metals and metalloids”) is a global concern due to their nonbiodegradable nature and persistence in different environmental compartments [1,2]. In addition, the presence of anomalous concentrations of trace metals in the environment is of great ecological importance, since these elements tend to bioaccumulate and biomagnify through the trophic chain [3,4,5]. The presence of anomalous concentrations of trace metals in the environment is mainly due to anthropogenic activities, such as mining or smelting, industry, agriculture and/or traffic [6,7,8,9]. Trace element inputs from sources may be stored in soils and sediments by various physicochemical processes, such as weathering, leaching and biological reactions, amongst others [10,11]. In the different environmental compartments, PTEs can exist in the form of different minerals but, also, as more labile compounds or species, the mobilities of which are dependent on the properties of each species and other environmental factors [12]. A knowledge of the total concentration of a given element in an environmental matrix such as soil or sediment allows a preliminary estimation of the potential risk to the environment [13]. However, the use of total concentrations may overestimate or underestimate the real risk to the environment [14]. A more realistic approach involves a determination of the proportion of the (bio) available fraction(s), which correspond to those that form compounds or species that are labile enough in the soil or sediments to be taken up by the organisms present [15]. In this respect, a range of chemical extraction procedures have been proposed to estimate the metal (bio) availability, with some based on the so-called sequential extractions [16,17,18] and others based on “selective extractions” [19,20,21]. Multistep sequential extraction procedures are widely used to study the mobility and potential (bio)availability of elements. This technique allows the differentiation of labile/mobile and residual/immobile fractions, and it has the advantage of allowing the different labile fractions to be characterized [22,23]. The data obtained provide information on the mobility, pathways or (bio) availability of the elements and any potential environmental hazards from soil or sediment samples [24,25]. The first sequential extraction procedure was developed by [16]. This method included five extraction steps to extract the following fractions: exchangeable (F1), bound to carbonates (F2), associated with iron and manganese oxyhydroxides (F3), bound to organic matter and sulfides (F4) and the residual fraction associated with recalcitrant minerals (F5).

However, in cases where the interest is in determining the available fraction that can be taken up by plants (plant-available fraction, [26], simple or selective extraction procedures are widely used [27]. This type of single-stage extraction is characterized by its simplicity and speed [28], and it provides important information about the potential risk of transfer between environmental compartments. A number of different extractants have been used with this aim, including water [29], ammonium acetate [30,31] or chelating agents such as ethylenediamine tetra acetic acid [32,33].

It is widely recognized that the total PTE concentrations in the soil overestimate the risk of phytotoxicity [34]. Several studies have shown that there is a low correlation between total PTE levels in soils and plant intakes [35,36]. This is a consequence of their different chemical forms and (bio) availability in soils: even the (bio) available fraction of PTEs in soils does not have a good correlation with the amount taken up by plants [37]. In fact, plants are able to modify the physicochemical properties of the soil and develop specific physiological strategies, such as excluding or accumulating plants [38,39].

In the work described here, sequential and selective extractions were performed on soils, sediments and mine tailings from an area affected by mining-related contamination by potentially toxic elements [13]. The studied area corresponds to the decommissioned mining area of Jebel Trozza, which is located in Central Tunisia. This was a medium-sized mine in which Zn was exploited from oxide ores hosted in a Mesozoic carbonate formation [40]. The mine activity extended from 1907 until the mine closure in 1937, and reclamation measures for the waste tailings have not been applied. Elmayel et al. described this area in detail [13], and they analyzed the total contents in the local tailings, soils and stream sediments. It was concluded that the area is highly contaminated in terms of total contents and that this contamination represents a hazard for the local water supply. It was proposed that new studies should be carried out with the aim of better evaluating the hazards.

On the basis of the above, the main objective of this research work was to assess the mobility of the potentially toxic elements present in the Jebel Trozza mine area. In particular, it was planned to determine the geographical variations in the mobility of the elements in order to identify criteria to understand the potential of these elements to change their (bio)availability during transportation processes in a semiarid context. The possibility of assessing the real potential risks associated with the presence of the decommissioned mine upstream of a reservoir used for irrigation was also considered.

## 2. Materials and Methods

### 2.1. Study Area

**Figure 1:**
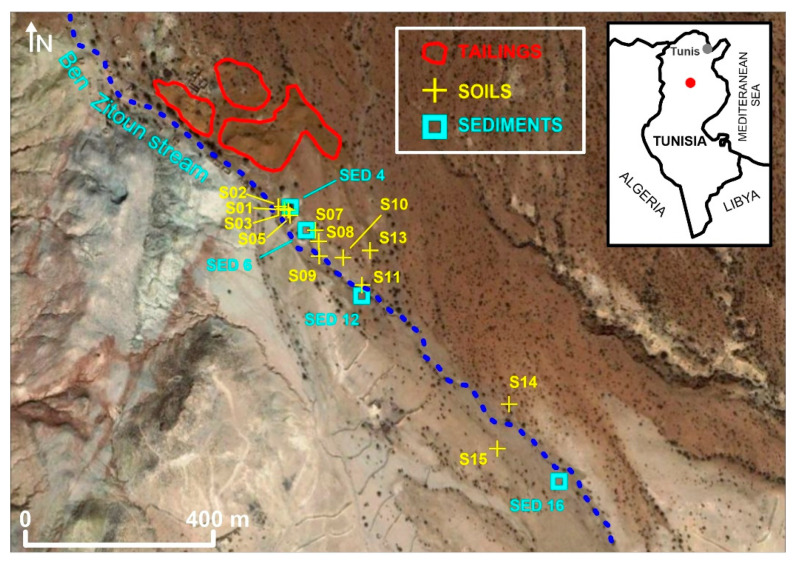
Locations of the study area (insert) and sampling sites. The source areas (tailings) are indicated.

The decommissioned Pb/Zn mine of Jebel Trozza is located approximately 15 km to the Southwest of Haffouz (Central-Northern Tunisia) and approximately 150 km SSW from Tunis (Figure 1). The area was exploited over thirty years, between the period 1907 to 1937, and after the cessation of the mining works, the area was totally abandoned without any restoration work, leaving different important environmental liabilities.

The area is distinguished by an intense arid climate with average annual precipitation of 100 mm, average annual temperature of 20 °C and potential evapotranspiration of 1750 mm·year^−1^ [41]. Due to the weather, a major part of the creeks is ephemeral, and only the Ben Zitoun main stream flows all year. The mining area is crossed by an ephemeral creek without name due to its scarce entity. When this creek flows, it is tributary of the Ben Zitoun before the irrigation El Houareb dam, which receives the solid and dissolved pollutions from the mining area. On the other hand, from a hydrogeological point of view, the Kairouan Plain aquifer is formed by Cretaceous carbonate reservoirs. The reservoirs are characterized by fractured limestones that suffer local dissolution and grading to a karstic system, which is frequent in Central Tunisia. The Jebel Trozza liabilities are the main cause of the deterioration of groundwater in the Kairouan Plain [42].

### 2.2. Sample Preparation and Analysis

Total concentrations of samples have already been reported [13]. A summary of the total concentrations from the selected samples for this research is shown in Table 1.

In this study, samples of approximately 1 kg were taken using a metal shovel, covering the shallowest 20 cm of the soil after removing the first layer of the surface soil (~2 cm). After that, samples were transferred to clean polyethylene bags and transported to the laboratory.

Extractions were performed on ten samples of soil surface horizons (codes from S02 to S15) with varying concentrations of PTEs, as well as on six samples of tailings clearly separated into two major types of mine wastes as a result of mineralurgical treatment: fine-grained residues (DF) and coarse-grained residues (DG) (codes DF and DG) and three samples of sediments (code SED) (Figure 1). The baseline soil sample (representing the local geochemical background) was obtained some 5 km away the mine area. Soil samples were taken following the stream direction, since the slope of the area has the same direction as the stream.

The sequential extraction method was performed to determine the exchangeable fraction (F1), the carbonate-bound fraction (F2), the oxyhydroxides fraction (F3), the organic fraction (F4) and the residual fraction (F5):

- F1: This fraction contains the metals that are considered to be adsorbed and not easily mobilized by ion exchange. A volume of 40 mL of magnesium chloride (1 M MgCl_2_) was added to 1 g of soil sample that had been dried in the open air and sieved at 0.63 mm (pH = 7). The mixture was stirred for one hour and then centrifuged at 3000 rpm for 20 min. The supernatant was recovered and filtered through a cellulose acetate membrane with a porosity of 0.45 μm and then stored prior to analysis by Inductively Coupled Plasma Optical Emisión Spectrometry (ICP-OES). The residue was stored for the remainder of the protocol.
- F2: This fraction corresponds to the acid-soluble fraction, and it combines the precipitated or coprecipitated metals with the natural carbonates, which dissolve on decreasing the pH. Besides, 8 mL of CH_3_COONa (1 M) at pH = 5 (adjusted with CH_3_COOH (1 M)) was added to the residue from step 1. The mixture was shaken for 5 h. The extract was recovered by the same procedure as in the previous step.
- F3: This fraction was obtained from the residue from the carbonated fraction (F2) by treatment with 20 mL of hydroxylamine chloride (0.04 M) in 25% acetic acid at 96 °C for 6 h.
- F4: This fraction was obtained by treatment of the residue from the preceding extraction with 3 mL of HNO_3_ (0.02 M) and 5 mL of H_2_O_2_ (30%) (pH = 2 with HNO_3_) at 85 °C for 2 h, 3 mL H_2_O_2_ (pH = 2 with HNO_3_) at 85 °C for 3 h and 5 mL CH_3_OONH_4_ in 20% (v/v) HNO_3_ diluted to 20 mL, with shaking for 30 min.
- F5: The residual fraction corresponds to recalcitrant or refractory minerals, usually with three metal ores present in the deposit, and this was obtained by adding hydrofluoric acid, hydrochloric acid and nitric acid to the residue from the preceding extraction. The extract was recovered by the same procedure as in the previous step.

The contents of the elements were quantified using ICP-OES (with plasma coupled by induction).

Sequential extraction data were obtained for soil and sediment samples along two transects, both starting at the edge of the tailings and running approximately parallel one to the other in a southerly direction. In the case of the soil samples, the transect had a N160ºE orientation, and for the sediment samples, the transect followed the course of an unnamed stream with a similar direction, which was a tributary of the Ben Zitoun stream. The outflow of the mine stream to the Ben Zitoun stream occurred at the so-called El Houareb Dam, which was originally built to provide drinking water for the Sahel Region but is now used for irrigation. Selective extractions also were carried out as follows:

The Environmental Protection Agency (EPA) method 1312 (synthetic precipitation leaching procedure) [43] consists of forming a suspension with 5 g of soil (<2 mm) and 100 mL of an H_2_SO_4_/HNO_3_ (60/40 weight/percent mixture) solution, adjusted to pH 4.2 ± 0.05. The different samples were placed in a thermostatic bath with stirring at 30 rpm for 18 h at room temperature. The supernatant was filtered through fiberglass syringe filters with a pore size of 0.70 μm. This procedure gave the soluble fraction in an oxidizing acidic medium that is capable of simulating acid mine drainage conditions [44,45].

Interchangeable species method: Extractions with 1-M ammonium acetate (NH_4_Ac) were carried out at pH 4.75 by the addition of glacial acetic acid to 5 g of the soil. One-hundred milliliters of the solution was shaken for two hours at 50 rpm at room temperature. The extracts were filtered through Teflon syringe filters (Sygma Aldrich, St Louis, MO, USA) (0.45 µm pore size). The resulting extract was more related to oxidizable mineralogical species, such as the fraction bound to organic matter and some sulfides [45,46].

All extracts were refrigerated until further analysis by ICP-MS (ICP-MS Thermo Electron XSeries II, Thermo Fisher Scientific, Bremen, Germany). For the analysis, different standards and calibration targets were prepared according to the matrix of extracts to be analyzed, and random duplicates were made.

## 3. Results and Discussion

### 3.1. Sequential Extraction Rsults

#### 3.1.1. Tailings Results

**Figure 2:**
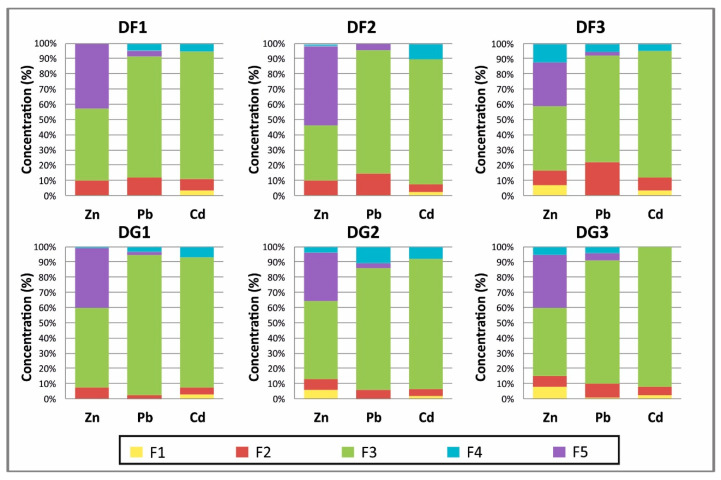
Proportions of the fractions upon sequential extraction of the tailing samples (DF-DG). DF—fine-grained residues; DG—coarse-grained residues; F—fractions extracted (F1, F2, F3, F4 and F5).

Analytical results for the various elements studied are presented in percentage diagrams (Figure 2). Sequential extractions on tailings (Supplementary Table S1) show that the highest percentages of Zn, Pb and Cd are related to the oxyhydroxides fraction (F3), with values ranging from 36.4% to 52.6% for Zn, from 69.9% to 92.3% for Pb and 82.1% to 92.14% for Cd. It can therefore be stated that there is a high affinity of Zn, Pb and Cd with the oxyhydroxides fraction.

The residual fraction (F5) fixes a less significant proportion than F3 for Zn, except for sample DF2 (F3 = 51.7%), which suggests a higher presence of recalcitrant minerals such as sulfides in this sample. In the case of Pb, F5 contains a lower proportion of this metal, with values ranging from 1.5% to 4.6%, while Cd is below the limit of detection (0.03 mg·kg^−1^). The carbonate fraction (F2) fixes a larger proportion of Pb than Zn and Cd, with percentages of 22%, 10% and 8.5%, respectively. In relation to carbonate, several authors have shown that Pb precipitates as Pb carbonates (mainly cerussite, PbCO_3_). According to [47] cerussite is the most stable supergene Pb mineral, and this results in reduced Pb mobility [48]. The organic fraction (F4) contains relatively small percentages of metals, but the values reach 11% for Pb, 12% for Zn and 10% for Cd.

Similarly, the exchangeable fraction (F1) fixes small proportions of the analyzed PTEs. F1 is the least-abundant fraction for Pb when compared to the other fractions, with percentages that vary between 0.03% and 1%, while, for Zn and Cd, the values are in the ranges 0.05–8% and 1–3%, respectively. Small proportions of PTEs are related to the exchangeable fraction. In similar studies reported by [49,50], the percentages of Zn and Pb were below 1%.

#### 3.1.2. Soil Samples

**Figure 3:**
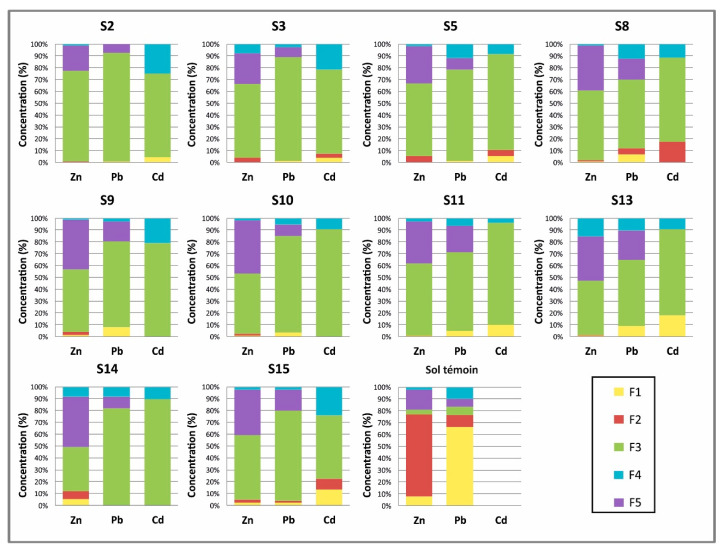
Proportions of the fractions upon sequential extraction of the soil samples (S). F—fractions extracted (F1, F2, F3, F4 and F5).

The distribution of the analyzed elements in the different fractions is variable in the contaminated soils of the Djebel Trozza mine area (Figure 3). However, as a general rule, the fraction associated with the oxyhydroxide fraction (F3) is the most important (Supplementary Table S2), except for the S11 sample, where the percentage of Zn related to the residual fraction (F5) is greater. The percentages in F3 vary between 37% and 77% for Zn, 55% and 92% for Pb and 53% and 90% for Cd. The residual fraction (F5) contains high percentages of Zn, with values up to 44% (sample S8). In the case of Pb, the proportion fixed by this fraction is less abundant (4–25%), whereas, for Cd, the percentages fixed by the residual fraction were below the detection limit in the analyzed samples. The exchangeable fraction (F1) (the most mobile and (bio)available fraction in the soils) has low proportions of Zn, Pb and Cd, with values in the ranges 0.1–2%, 0.5–8% and 2–5%, respectively.

In the case of the carbonate fraction (F2), the proportions of Zn, Pb and Cd bound to this fraction are low: for Zn, the percentages are in the range 0.13–4%, while, for Pb and Cd, the percentages were below the detection limits in some samples but reached up to 5% to 17%, respectively.

Finally, the amounts of Zn, Pb and Cd bound to the organic fraction (F4) are not negligible; the proportions vary between 1% and 15% for Zn, 3% and 23% for Cd and 0.05% and 12% for Pb.

The results of the sequential extraction of the agricultural soil are also included in Figure 3 (denoted as the baseline in Figure 3). These soils were sampled outside the mine area around 6 km from the mine site. Large differences can be observed in the fractionation of the different phases, with the majority being due to phase changes in the mining materials. These differences concern F3 and F5 and the baseline soil F2 in terms of Zn and F1 in Pb. Surprisingly, F5 contains similar proportions of these elements in mining and baseline soils, although the total concentrations are different. A similar pattern can be observed for F4 in Pb, with comparable values in many mining and baseline soils. Unfortunately, data could not be obtained for Cd fractionation, because the baseline sample did not surpass the detection limits for this element. In summary, mining soils have a higher proportion of PTEs in immobile (F5) or poorly mobile (F3) phases, while the reference soil, which has a much lower PTE content, has these elements in more soluble and available phases (F1 and F2).

#### 3.1.3. Sediment Samples

**Figure 4:**
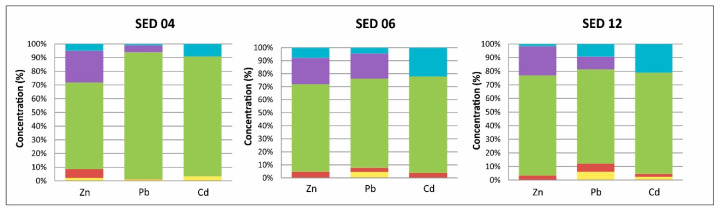
Proportions of the fractions upon sequential extraction of the sediment samples. SED—sediment sample.

In order to study the behavior of PTEs in sediments, sequential extraction using the Tessier method (Tessier et al., 1979) was performed on three selected samples. The results (Supplementary Table S3) indicate that the fraction of Fe and Mn hydroxides (F3) fixes the majority of the PTEs; this fraction accounts for up to 68% of Zn, 78% of Cd and 76% of Pb (Figure 4). The residual fraction also contained significant proportions of Zn and Pb, albeit at lower percentages than the hydroxides fraction (22% for Zn and 11% for Pb), and cadmium was not detectable in this fraction. The organic fraction fixed 17% of Cd, whereas the percentages of Zn and Pb did not exceed 5%. PTEs linked to the carbonate fraction are low, with values of 4% for Zn, 3% for Pb and 2% for Cd. Similarly, in the case of the exchangeable fraction, only a small proportion of the PTEs are linked to this fraction (1% for Zn, 4% for Pb and 2% for Cd).

#### 3.1.4. Comparison between Tailings, Soils and Sediments Fractionations

The speciation of PTEs in the contaminated soils of Jebel Trozza showed that the elements studied had an affinity for the hydroxides fraction. Furthermore, a high proportion of the Zn and Pb is related to the residual fraction (F5), but Cd could not be detected in this fraction.

The low percentages of PTEs in the water-soluble (F1), carbonate (F2) and organic (F4) fractions may be related to the occurrence of reabsorption phenomena during extractive reactions. The phenomenon of reabsorption generally depends on the chemical element, the chemical properties of the extractant and the conditions during extraction and the nature of the sample [51,52].

Despite the small proportions of exchangeable fraction detected (F1), these may be associated with other fractions, such as organic matter or iron oxides, or they may transfer to plants [50].

**Figure 5:**
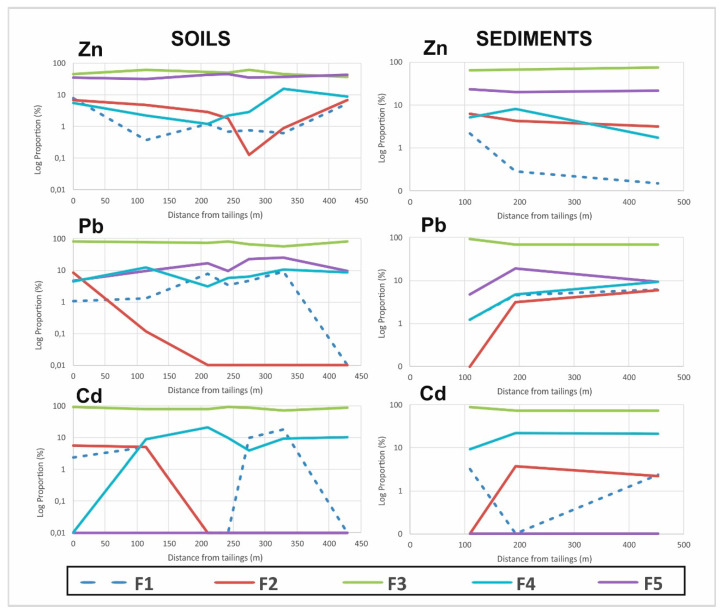
Evolution of the phase fractionation along the longitudinal transects. The limit of the tailings is taken as the starting point. F—fractions extracted (F1, F2, F3, F4 and F5).

It can be seen along both transects that the most abundant fraction (F3) evolves without significant changes, with similar starting and destination points in the case of the soils for the three elements considered and showing a decreasing tendency on moving away from the tailings in the cases of Pb and Cd in the sediments. In this case, significant differences were not observed in the transport mechanisms between soils and sediments, since the climate—with rare torrential rain—produces a similar transport in soils and sediments (the river remains dry between rainy periods). This could be the reason why a similar pattern of coincidence is observed between the initial and final levels in the F5 fraction for Zn and Pb in the transect. Physicochemical mechanisms of transport do not transform the main fractions (F3 and F5) along the transects. However, such mechanisms do produce a chaotic evolution pattern for F1, F2 and F4 for Zn and a dramatic decrease in fractions F1 and F2 for Pb in soils. In sediments, however, the trend is opposite, with the mobile fractions F1 and F2 showing a decrease for Zn and stability for Pb (Figure 5). These differences in the evolution of F1 and F2 in terms of Zn and Pb could be significant, especially given the contrasting behavior of these two elements due to the differences in their ionic potentials as Pb^2+^ and Zn^2+^, i.e., extremely high for Zn and extremely low for Pb. As a consequence, Pb will remain close to the tailings, while Zn will move large distances from the tailings [53] in the river system. In the case of sediments, it is necessary to take into account the ionic stability of the studied elements (Zn and Pb). In freshwater systems, Zn^2+^ will be the most stable species below pH 8, while Pb will be complexed by carbonate species (Pb(CO_3_)_2_^2−^) at pH 6–8 [54]. This fact means that the more mobile fractions of Zn will decrease (F2) or even disappear (F3) at some point in the sediment transects, while the more mobile fractions of Pb (F2 and F3) will remain in similar proportions throughout transport in the sediments. The question arises as to which processes explain the opposite behavior of these labile fractions (F1 and F2) in soils. Of particular interest is the disappearance of the carbonate-bound fraction (F2) of Pb in a carbonate environment. This finding can be explained, because Pb^2+^ in soils with a pH close to 8 (typical of semiarid and arid regions) will first form Pb oxides, followed by the formation of cerussite (PbCO_3_) if the conditions are favorable. In the same environment, Zn^2+^ may form smithsonite (ZnCO_3_) or hydrozincite (Zn_5_(CO_3_)_2_(OH)_6_), and this could explain the retention of Zn levels in the F2 fraction in the soil transect. Another interesting aspect is the maintenance of F4 proportions along the Zn and Pb transect, although Pb has much higher affinity than Zn for binding with organic matter [55,56]. However, the soil organic matter (SOM) can act as a chelator, and this would immobilize Zn and Cd, in addition to Pb [57]. In fact, the F4 fraction for Cd in soils experiences a spectacular increase very soon after moving away from the tailings—a finding that confirms the above hypothesis.

Physicochemical mechanisms of transport do not produce significant transformations in the main fractions along transects, but for Cd, the residual fraction (F5) does not appear as a major fraction. Cd is normally included in the structure of sphalerite as an isomorphous impurity [58] in this type of mineralization, but if this was the case, a parallelism would be observed between both elements, at least in the F5 fraction. The most feasible explanation is that Cd and Zn appear as parts of different mineral phases with different solubilities. In this carbonated environment, Cd can be present in the form of otavite, a Cd carbonate frequently associated with the appearance of other Zn carbonates like smithsonite. Cd carbonates are insoluble in water but are soluble in weak acids, as in fraction F3, which was extracted with hydroxylamine and gave rise to the highest level of Cd. The evolution of other phases includes an increase in F4 in soils and sediments downstream and a chaotic variation in the F1 fraction.

### 3.2. Selective Extractions

In order to evaluate the (bio)available fraction suitable for plant uptake, two selective extraction methods were performed: extraction with ammonium acetate and extraction with H_2_SO_4_/HNO_3_ (EPA method, synthetic precipitation leaching procedure) [43]. According to [45], these two methods seem to be the most effective for estimating the (bio) available fraction of PTEs.

The results of the H_2_SO_4_/HNO_3_ extractions showed low-to-negligible contents of PTEs, and it can therefore be concluded that this extraction protocol is not suitable for the samples from the study area. The extractions with ammonium acetate (NH_4_Ac) were carried out at pH 4.75, and this method is considered to represent the exchangeable fraction [45,59]. At pH 4.75, the extract obtained has also been described as a fraction associated with the oxidizable mineralogical species present in the soil (e.g., organic matter and certain sulfides) [46].

#### 3.2.1. Tailings Results

The chemical fractionation results obtained for the three elements studied (Pb, Zn and Cd) in the different tailings samples from Jebel Trozza obtained by the selective extraction procedure with NH_4_Ac are presented in Table 2. This method releases not only the exchangeable fraction (F1) but, also, the fraction associated with carbonates (F2) [23].

Based on the results, for both types of mine waste, the quantity of PTEs present in the interchangeable fraction is very low, i.e., below 1.5% for Zn, 0.5% for Cd and 2.5% for Pb. On comparing the percentages of the three elements studied, the quantity of Pb extracted is more significant than those of Zn and Cd.

#### 3.2.2. Soil Results

**Figure 6:**
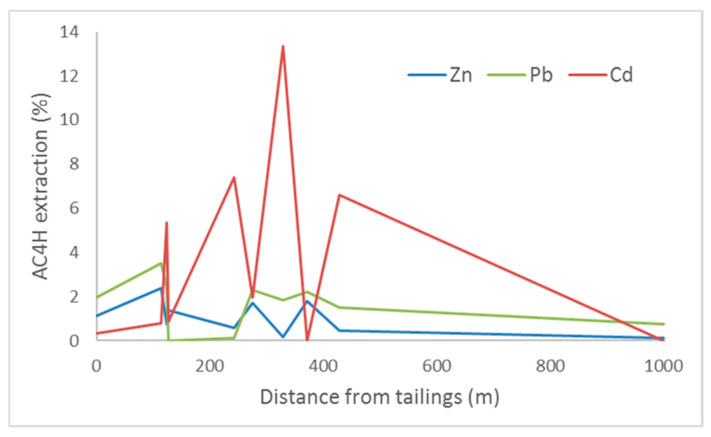
Evolution of the (bio)available fractions of Pb, Zn and Cd in the soil samples.

The results for the soil samples show that, for Zn, the (bio)available fractions are low when compared with the total content, i.e., 0.6%, except for S3 and S15, which were above 2%. In the case of Pb, the percentages obtained ranged from 0.2% (for the S8 sample with the lowest concentration in Zn) to 3.5% (for the S15 sample). These percentages are higher than those recorded for Zn. Comparison of the (bio)available Cd levels with the total levels shows that the (bio)available fraction is significant; the percentages are negligible in some samples, but they exceed 13% in others. Generally, the percentage of (bio)available Cd is much higher than for Pb and Zn (Figure 6). As far as mobility is concerned and, thus, the availability of different metals, the sequence follows the order Cd > Pb > Zn. It can be seen from Figure 6 that the evolution of the bioavailability data for these three elements starts in the order Pb > Zn > Cd in the tailings. However, at a very short distance from the tailings—and with the elements already incorporated into the soils—this order changes to Cd > Zn > Pb. At a distance of 200 m from the tailings, the order changes again to Cd > Pb > Zn. It seems that the different compounds in which Cd is present (described previously based on the sequential extraction data) become more available, or (bio)available, in the edaphic dynamics of a soil in a semi-arid climate. Transportation processes do not seem to alter this situation, as changes in magnitude were not observed up to 1000 m away, although a marked increase in the bioavailability of Cd was observed in the vicinity of the tailings (between 200 and 400 m), which could be problematic in terms of the risk to people or the food chain. This maximum in the Cd (bio)availability coincides with an increase in the organic fraction (F4) in the sequential extraction. It seems clear that soil organic matter plays an important role in this increased availability by fixing PTEs in the organic soil matrix less strongly for Cd than for Zn and Pb. These data are consistent with those published by Antoniadis and Alloway [60], who reported that increasing amounts of SOM increase the (bio) availability of PTEs, especially for Cd and Pb. At the end of the transect, the bioavailability of the PTEs is markedly lower than in the vicinity of the tailings. Therefore, the edaphic and transport processes reduce the risk derived from the presence of PTEs (especially Cd) in these mining-derived materials.

**Figure 7:**
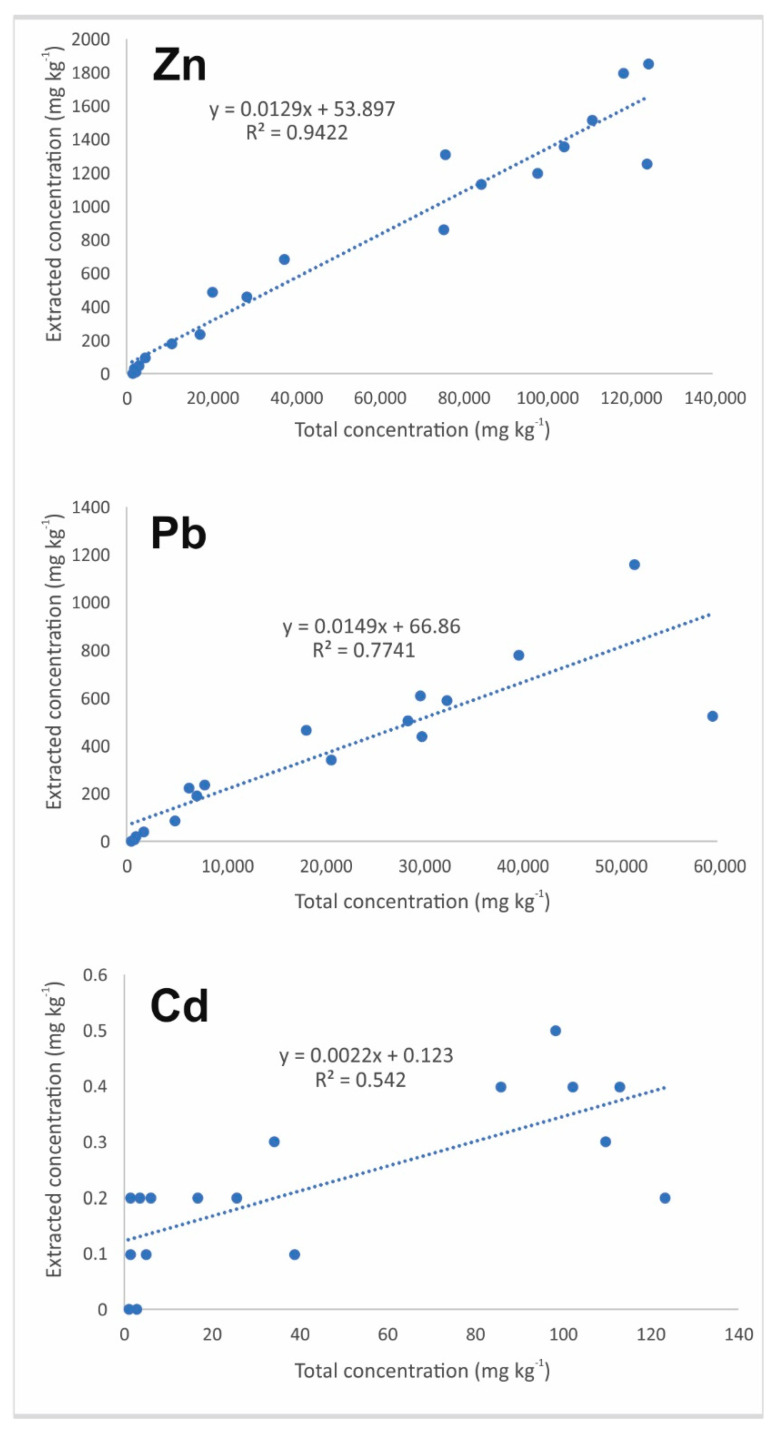
Correlations between the total and extracted concentrations of Pb, Zn and Cd in the soil.

A basic Pearson’s correlation study was performed in order to understand the relationships between the total and extractable concentrations. It can be seen from Figure 7 that there is a good correlation between the concentrations in the soil and those extracted in the (bio) available fraction for Zn and Pb, with *R^2^* = 0.942 (*p* < 0.05) and 0.774 (*p* < 0.05), respectively.

The correlation between the total concentration of Cd and that in the (bio)available phase is not as good, but it is still significant (*R^2^* = 0.542; *p* < 0.05). This finding is a good indication of the homogeneity of the extractability of Zn and Pb, whereas, for Cd, it is a good indication of a very variable extractability.

The results of this study show that, in terms of mobility and (bio)availability, the PTEs are not mobile or have very low mobility. Indeed, only a small proportion of these elements were solubilized in H_2_SO_4_/HNO_3_ (EPA method, synthetic precipitation leaching procedure), and this indicates that their passage into a solution under natural conditions is probably very low. In addition, the extraction of interchangeable species with ammonium acetate at pH = 4.75 shows the presence of considerable percentages of Cd, Pb and Zn in this phase, thus suggesting the potential availability of these elements.

## 4. Conclusions

The sequential extraction of tailings, soils and sediments from a decommissioned Zn mine area show that the highest percentages of Zn, Pb and Cd are related to the oxyhydroxides fraction (F3), while the residual fraction (F5) fixes a minor proportion. These results suggest a higher presence of recalcitrant minerals in F5. In contrast, the carbonate-bound (F2), organic (F4) and exchangeable (F1) fractions appear to be minor sources of PTEs.

Physicochemical mechanisms of transport do not transform the main fractions (F3 and F5) along the transects. Instead, the more mobile fractions of Zn decrease (F2) or even disappear (F3) at some point in the sediment transects, while the more mobile fractions of Pb (F2 and F3) remain in similar proportions throughout transport in the sediments.

Sediments have shown a decrease of Zn, which is surprising in a carbonate environment. This finding can be explained, because Zn^2+^ can form smithsonite (ZnCO_3_) or hydrozincite (Zn_5_(CO_3_)_2_(OH)_6_), which is consistent with the presence of Zn in the F2 fraction in the soil transect, while Pb and Cd may appear as different mineral phases.

Another interesting finding is the retention of F4 proportions along the soil transect for Zn and Pb, while, for Cd, this fraction increases significantly—which is explained by the role of SOM as a chelator for these PTEs.

The relatively high mobility found for Cd in soils indicates a potential risk for agriculture, since its (bio)availability increases with distance. Furthermore, the increase in this parameter associated with the aquatic transportation of this element highlights the possible input into a water mass that could be used in agriculture. Further research is required to confirm the hypotheses outlined above, particularly at the reservoir downstream from the mine area.

## Figures and Tables

**Table 1:** Total concentrations of the potentially toxic elements (PTEs) in the selected tailings, sediments and soil samples.

Materials	Samples	Zn (mg·kg^−1^)	Pb (mg·kg^−1^)	Cd (mg·kg^−1^)
Tailings	DF1	3795	1865	25
	DF2	3815	1675	35
	DF3	4235	3740	40
	DG1	3720	2110	50
	DG2	4855	3425	15
	DG3	5135	2395	70
Sediments	SED4	1445	1770	20
	SED6	730	1870	40
	SED12	1645	1530	15
Soils	S2	955	860	10
	S3	1005	1395	9
	S5	1020	960	10
	S8	130	280	5
	S9	325	495	7
	S10	305	260	5
	S11	215	220	5
	S13	1080	1825	12
	S14	855	1075	10
	S15	245	370	4

Abbreviations: DF—fine-grained residues; DG—coarse-grained residues; SED—sediment; S—soil.

**Table 2:** Contents of the (bio)available fractions of the potentially toxic elements (PTEs) in coarse mine tailings (DG) and fine grainsize tailings (DF).

Samples	Concentrations (mg·kg^−1^)		
	Zn	Cd	Pb
**DG1**	1516 (1.36%)	0.4 (0.39%)	508 (1.77%)
**DG2**	1800 (1.51%)	0.4 (0.35%)	442 (1.47%)
**DG3**	1854 (1.48%)	0.4 (0.35%)	610 (2.04%)
**DG4**	1356 (1.29%)	0.4 (0.37%)	340 (1.62%)
**DG5**	1200 (1.22%)	0.3 (0.27%)	466 (2.54%)
**DF1**	1136 (1.33%)	0.4 (0.46%)	778 (1.94%)
**DF2**	858 (1.13%)	0.5 (0.5%)	1162 (2.24%)
**DF3**	1306 (1.71%)	0.5 (0.43%)	526 (0.88%)
**DF4**	1252 (1.005)	0.2 (0.16%)	590 (1.81%)

## Supplementary Materials

The following are available online at https://www.mdpi.com/1660-4601/17/14/4912/s1, Table S1. Concentrations of PTEs in each sequential extraction step for tailing samples and recovery percentage respect to the total, Table S2. Concentrations of PTEs in each sequential extraction step for soil samples and recovery percentage respect to the total, Table S3. Concentrations of PTEs in each sequential extraction step for sediment samples and recovery percentage respect to the total.
