# Supplementary material for: Evolution of the Speciation and Mobility of Pb, Zn and Cd in Relation to Transport Processes in a Mining Environment
Source: Int J Environ Res Public Health. 2020 Jul 8;17(14):4912. doi: 10.3390/ijerph17144912 (PMC7400175; doi:10.3390/ijerph17144912)
Supplement: Supplementary file 1 [file ijerph-17-04912-s001.pdf]

International Journal of
Environmental Research and Public Health

MDPI

Supplementary Table S1. Concentrations of PTEs in each sequential extraction step for tailing samples and recovery percentage respect to the total.

Tailing samples	PTEs	F-E ($\mu g \cdot g^{-1}$)	F-C ($\mu g\ g^{-1}$)	F-Ox ($\mu g\ g^{-1}$)	F-Orga ($\mu g\ g^{-1}$)	F-Resi ($\mu g\ g^{-1}$)	Recovery (%)
DF1	Zn	2.98	372.05	1788.26	14.47	1614.20	99.92%
	Pb	4.52	216.22	1485.25	93.38	63.73	99.90%
	Cd	0.70	1.60	17.57	1.16	<DL *	84.10%
DF2	Zn	2.06	369.94	1389.79	76.63	1974.07	99.93%
	Pb	2.37	238.03	1362.88	11.20	59.91	99.96%
	Cd	0.72	1.67	27.63	3.62	<DL *	96.12%
DF3	Zn	2.04	393.78	1788.29	525.62	1228.29	99.99%
	Pb	4.53	821.45	2612.69	198.64	99.25	99.91%
	Cd	1.20	2.91	29.15	1.79	<DL *	87.62%
DG1	Zn	2.53	264.51	1956.57	28.87	1465.31	99.94%
	Pb	0.84	48.83	1947.03	78.36	33.62	99.94%
	Cd	1.27	2.09	38.59	3.14	<DL *	90.19%
DG2	Zn	2.17	344.04	2489.24	196.79	1527.08	99.90%
	Pb	1.27	204.25	2732.87	375.58	106.66	99.87%
	Cd	0.23	0.54	10.77	1.00	<DL *	83.58%
DG3	Zn	2.37	352.06	2314.53	282.18	1772.39	99.99%
	Pb	25.14	206.02	1943.18	106.18	110.40	99.83%
	Cd	1.55	3.56	59.93	<DL *	<DL *	92.91%

* < DL—Below Detection Limit; DF—fine-grained residues; DG—coarse-grained residues; F-E—exchangeable fraction; F-C—carbonate-bound fraction; F-Ox—oxyhydroxides fraction; F-Org—organic fraction; F-Resi—residual fraction.

Supplementary Table 2. Concentrations of PTEs in each sequential extraction step for soil samples and recovery percentage respect to the total.

Soil samples	PTEs	F-E ($\mu g \cdot g^{-1}$)	F-C ($\mu g\ g^{-1}$)	F-Ox ($\mu g\ g^{-1}$)	F-Orga ($\mu g\ g^{-1}$)	F-Resi ($\mu g\ g^{-1}$)	Recovery (%)
S2	Zn	2.93	4.16	731.87	12.57	201.64	99.81%
	Pb	4.83	0.92	788.51	0.51	63.37	99.78%
	Cd	0.42	<DL *	6.57	2.37	<DL *	93.64%
S3	Zn	4.20	35.16	623.63	80.27	260.37	99.86%
	Pb	17.91	1.65	1221.32	39.09	113.03	99.86%
	Cd	0.27	0.27	5.07	1.54	<DL *	71.48%
S5	Zn	3.70	49.42	625.37	22.44	315.41	99.64%
	Pb	12.51	1.11	735.84	114.66	92.71	99.67%
	Cd	0.41	0.40	6.41	0.69	<DL *	79.01%
S8	Zn	0.95	1.60	74.25	1.86	47.38	96.95%
	Pb	19.55	13.36	160.53	34.58	50.01	99.30%
	Cd	<DL *	0.63	2.59	0.42	<DL *	72.94%
S9	Zn	3.79	9.31	169.10	3.92	136.10	99.14%
	Pb	38.03	<DL *	358.53	15.63	80.26	99.48%
	Cd	<DL *	<DL *	4.13	1.09	<DL *	74.61%
S10	Zn	2.03	5.54	154.08	6.68	134.27	99.21%
	Pb	8.83	<DL *	211.11	14.47	24.56	99.60%
	Cd	<DL *	<DL *	3.58	0.38	<DL *	79.28%

S11	Zn	1.63	0.27	130.06	6.14	76.16	99.66%
	Pb	10.24	<DL *	144.83	13.75	48.02	98.56%
	Cd	0.40	<DL *	3.55	0.16	<DL *	82.24%
S13	Zn	6.57	9.37	489.69	165.17	405.79	99.68%
	Pb	161.15	<DL *	1015.46	188.77	456.36	99.82%
	Cd	1.86	<DL *	7.59	0.98	<DL *	86.24%
S14	Zn	44.93	56.91	318.88	73.64	359.49	99.87%
	Pb	<DL *	<DL *	878.80	91.09	103.45	99.85%
	Cd	<DL *	<DL *	8.28	0.96	<DL *	92.41%
S15	Zn	5.57	6.57	131.15	6.17	92.53	98.77%
	Pb	8.09	5.50	279.47	9.32	63.33	98.84%
	Cd	0.44	0.31	1.78	0.80	<DL *	83.35%

* < DL—Below Detection Limit; S—soil. F-E: exchangeable fraction; F-E: exchangeable fraction; F-C—carbonate-bound fraction; F-Ox–oxyhydroxides fraction; F-Org—organic fraction; F-Resi—residual fraction.

Supplementary Table 3. Concentrations of PTEs in each sequential extraction step for sediment samples and recovery percentage respect to the total.

Sediment samples	PTEs	F-E (μg·g^{-1})	F-C (μg g^{-1})	F-Ox (μg g^{-1})	F-Orga (μg g^{-1})	F-Resi (μg g^{-1})	Recovery (%)
Sed4	Zn	31.19	91.36	913.53	73.92	331.79	99.78%
	Pb	21.16	1.45	1636.50	21.36	84.95	99.74%
	Cd	0.60	<DL *	16.16	1.70	<DL *	92.30%
Sed6	Zn	2.09	31.44	490.18	58.25	146.31	99.76%
	Pb	86.47	58.87	1278.00	88.28	355.99	99.87%
	Cd	<DL *	1.41	28.10	8.41	<DL *	94.83%
Sed12	Zn	2.44	52.02	1206.42	27.94	352.65	99.79%
	Pb	94.64	90.58	1059.71	142.29	142.34	99.97%
	Cd	0.32	0.29	9.98	2.85	<DL *	89.60%

* < DL—Below Detection Limit; SED—sediment; F-E—exchangeable fraction; F-C—carbonate-bound fraction; F-Ox—oxyhydroxides fraction; F-Org—organic fraction; F-Resi—residual fraction.
